# The Application of Flipped Classroom Combined With Locus of Control Analysis in Lean Entrepreneurship Education for College Students

**DOI:** 10.3389/fpsyg.2020.01587

**Published:** 2020-07-22

**Authors:** Yueqi Qin, Runyu Yan, Youxia Sun

**Affiliations:** ^1^The Institute of Curriculum and Instruction, East China Normal University, Shanghai, China; ^2^School of Law, East China Normal University, Shanghai, China; ^3^School of Foreign Languages, East China Normal University, Shanghai, China

**Keywords:** flipped classroom, locus of control, deep learning, lean entrepreneurship education, entrepreneurial motivation

## Abstract

This paper aims to explore the status quo and basic laws of entrepreneurship education at the stage of social development in China, thereby providing a theoretical basis and practical guidance for the cultivation of innovative and entrepreneurial talents in colleges. First, a college entrepreneurship education model based on lean entrepreneurship theory and flipped classroom was established to provide the development of entrepreneurship education with a theoretical framework while improving the students’ autonomous learning ability. Based on the theoretical basis of the influence of the locus of control on entrepreneurial motivation in the entrepreneurial process of college students, the students who participated in the basic education of entrepreneurship among the sophomores in the first semester of the 2018–2019 academic year of a college in Shanghai were selected as the research object. Then, the effect of lean entrepreneurship education under the flipped classroom mode was analyzed. Before the start of the entrepreneurial basic education course, there was no difference between the pretest scores of the research group and the control group students (*P* > 0.05). After the course, there was no difference between the posttest scores of the two groups of students (*P* > 0.05). It can be confirmed that, based on the flipped classroom education mode of halving the actual number of lectures by teachers, the effect of basic education on entrepreneurship for students is not different from the traditional teaching effect. Based on the flipped classroom mode, the number of people who have reached the level of “understanding” of the text target task is the highest, and the number of people who can reach the “comprehensive application” level of the high-order target is almost 0. It means that the realization of the high-order target still needs to be strengthened. Lean entrepreneurship education mode is based on lean iteration, which is conducive to promoting the development of entrepreneurship education in China. Therefore, the application of flipped classroom combined with locus of control analysis in lean entrepreneurship education for college students can ideally achieve the goal of deep learning, which is greatly significant for improving the effectiveness of entrepreneurship education.

## Introduction

College entrepreneurship has gradually received attention and recognition from the earliest novel employment situation, and it has become a new way to solve employment problems after college students enter society. Employment driven by entrepreneurship can fundamentally solve the problem of difficult employment for college students and alleviate the pressure on employment across the country. In addition, the vitality of entrepreneurs will lead to an increase in employment (Souza, 2020). China has entered the era of “mass entrepreneurship and innovation,” and the current society encourages college students to start businesses. From the perspective of college students themselves, the main driving force for entrepreneurship is to seek the realization of self-worth. Through entrepreneurship and entrepreneurial practice, they can fully mobilize their subjective initiative, change their employment mentality, and learn to self-regulate and control (Frederiksen and Brem, 2017). From the perspective of social development, more and more college students are beginning to have a clearer intention to invest in the entrepreneurial boom. It can not only provide college students with more theoretical and practical opportunities but also have an important role in promoting social and economic development. As a college that guides students to diversify their entrepreneurial thinking, entrepreneurship education itself is a course with innovative awareness requirements. Also, the entrepreneurial basic course has now occupied a certain proportion of college education courses. Only by integrating advanced entrepreneurial innovation modes and concepts into the basic courses can it play a practical role in education and teaching (Yang et al., 2019). Therefore, how to effectively integrate the entrepreneurial mode of lean entrepreneurship with the basic courses of entrepreneurship in colleges has become a hot issue in the field of entrepreneurship education.

At this stage, entrepreneurship education in China’s colleges is still in the initial exploration stage. A new set of theories and methods is urgently needed to lead the development in the field of entrepreneurial basic education. The theory of innovative development methods represented by lean thinking has begun to be studied and widely applied (Harms and Schwery, 2020). Lean entrepreneurship provides a theoretical basis for the development of innovative activities. Through application of lean entrepreneurship, entrepreneurial basic education can also be improved in experiments. In the 21st century informatization era, the teaching method, which is based on direct instruction by teachers, has caused students’ passive and negative learning attitudes to a certain extent. After the flipped classroom was proposed as a new teaching concept, it not only put forward the requirements for students’ learning depth but also provided time and space conditions for colleges to develop deep learning (Lichtenthaler, 2020). The flipped classroom advocates the principle of “pre-class knowledge transfer and in-class knowledge internalization” and proposes that students may independently acquire key extracurricular knowledge related to the course. Then, the existing problems are solved in the classroom and the knowledge is digested through discussion and communication. In the process of entrepreneurship education in colleges, students have an understanding and cognition of entrepreneurship through the flipped classroom. As a result, the auxiliary guidance in class makes the connection clearer between innovation and entrepreneurship with their development plan (Hwang and Shin, 2019).

In the process of carrying out lean entrepreneurship education in colleges, it is also important to judge the psychological state of students. In the process of entrepreneurship, college students can give full play to their subjective initiative and creativity, reflect their entrepreneurial ability, and ultimately realize their life value. Their self-efficacy in entrepreneurship is an important condition, leading the entrepreneurs to start a business and persist in their belief in entrepreneurship. Moreover, it will have an impact on entrepreneurial motivation, and locus of control as a key personal factor will also affect entrepreneurial motivation. Studies have shown that the more the score on the locus of control tends to internal control, the higher the score on self-worth. As the achievement motivation and self-efficacy of college students go higher, it means that they have had a comprehensive assessment of themselves and are fully prepared for entrepreneurship (Miao et al., 2019).

This paper aims to explore the status quo and the basic laws of entrepreneurship education at the stage of social development in China, providing a theoretical basis and practical guidance for the cultivation of innovative and entrepreneurial talents in colleges. This paper first established an entrepreneurship education mode in colleges based on lean entrepreneurship theory, thereby providing a theoretical framework for the development of entrepreneurship education. It is considered that the flipped classroom mode provides the possibility of achieving deep learning for students. Therefore, the flipped classroom mode was introduced in the process of entrepreneurship education, hoping to give students more autonomy and promote the realization of high-order entrepreneurship teaching goals.

## Literature Review

Entrepreneurship can be seen as a universal activity in a market economy, including definitions in both narrow and broad senses. Broadly speaking, it means starting a new business. Narrowly speaking, it means creating a new enterprise. In this paper, the entrepreneurship on a narrow level was explored, that is, innovative enterprises. In the entrepreneurial process, entrepreneurial intention is at the core of the entrepreneurial psychological process and is the best prediction indicator of entrepreneurial behavior. Only potential entrepreneurs with a certain degree of entrepreneurial intention are really likely to engage in entrepreneurial activities. In the exploration of the entrepreneurial intention of college students, Christian et al. believed that the entrepreneurial intention of college students refers to the possibility of actual entrepreneurship in the foreseeable future after graduation (Lüthje and Franke, 2003). In a series of follow-up surveys on college students’ entrepreneurial intentions, it is found that it is necessary to enhance college students’ entrepreneurial awareness and increase the entrepreneurial knowledge reserve as well as entrepreneurial motivation through systematic entrepreneurship education.

Introducing different operations in the innovation of teaching methods is an effective way to enhance the effect of entrepreneurship education, including “MOOC” and “flipped classroom.” Lean Startup, a specific application in the field of innovation and entrepreneurship, was first proposed by Silicon Valley entrepreneur Eric Ries in his book *Lean Startup* in 2012. After this, investor Blank suggested that entrepreneurship using lean startup methods would have a lower failure rate than the traditional methods. The method is greatly significant to the development of innovative economies in various countries (Blank, 2016). There are great prospects for applying the concept of lean startup to entrepreneurship education. Through the measurement and evaluation of each step as well as all the phased actions and progress, the understanding of the fact is obtained after the test. Mansoori (2017) explored the impact mechanism of lean startup guidelines on entrepreneurs based on the semi-structured in-depth interview design. He believes that entrepreneurs are learning, internalizing, and applying lean startup methods through alternative learning and learning in practice (Mansoori, 2017).

In general, colleges around the world have already started compulsory courses related to entrepreneurship in accordance with the requirements or regulations of the Ministry of Education. The basic education of entrepreneurship for college students is a part of general education and does not take the number and performance of founding companies as teaching goals. There are extremely uncertain delays in actual entrepreneurial behavior. Therefore, the investigation on entrepreneurship basic education from the perspective of individual intention is a more practical and feasible option. Integrating the concept of lean startup into the process of entrepreneurship education is suitable for high-tech or Internet entrepreneurship, as well as a wider range of fields. Therefore, under the entrepreneurial education mode of colleges based on lean startup theory, while improving students’ independent learning ability, it provides a theoretical framework for the development of entrepreneurship education.

## Materials and Methods

### The Influence of Locus of Control on Entrepreneurial Motivation

Entrepreneur psychology is a special psychological phenomenon manifested during the course of entrepreneurial behavior, that is, the mental state in which entrepreneurs regulate and control entrepreneurial behavior in entrepreneurial activities. In the process of entrepreneurship, college students can give full play to their subjective initiative and creativity, reflect their entrepreneurial ability, and ultimately realize their life value. In the process, they face pressure from various parties. Therefore, college entrepreneurs are required to be able to maintain good stress resistance at all times, facing difficulties and unknown risks in the process of entrepreneurship.

The concept of locus of control is widely used in the field of psychology. It was first proposed by Rotter in *Social Learning Theory* in 1953 (Euchner, 2018). Until now, however, no scholar has a clear definition of it. It is generally recognized as the cause of an individual’s behavioral outcome. The locus of control is a multidimensional concept, divided into internal control and external control. The former believe that productive behavior is attributed to one’s own efforts. The latter believes that the outcome of the event has nothing to do with one’s own efforts. The existing research results show that the motivation of entrepreneurs to pursue success has a significant positive correlation with the locus of control, while the motivation to avoid failure has a significant negative correlation with the locus of control. Students with a superior locus of control usually have a better psychological state. If they can internally attribute their control points well when individuals face success or failure results in the event, the psychological state will also show a good trend (Yordanova, 2017). From this perspective, the locus of control can help individuals understand the nature of things and enable them to better control emotions.

At present, most worldwide scholars’ research on entrepreneurial motivation is mainly focused on the entrepreneur, and there is less research on entrepreneurial motivation of college students. The employment situation of college students in China is relatively severe, and entrepreneurship is an effective way to alleviate employment pressure. Therefore, research on college students’ entrepreneurial motivation is of practical significance (Llamas and Fernández, 2018). The development and the conduct of entrepreneurial activities require greater courage and firm conviction. College students with internal control trend believe that their efforts can promote the process of entrepreneurship and complete entrepreneurial activities. They can also take full advantage of the uncertain risks and challenges in the entrepreneurial process. The locus of control has a regulating role in stimulating college students’ entrepreneurial motivation. The lower the score of the locus of control for college students, the stronger the internal control trend. Entrepreneurial self-efficacy has a significant stimulating effect on college students’ entrepreneurial motivation. The higher the score of the locus of control for college students, the higher the external control trend. The stimulating effect of entrepreneurial self-efficacy on its entrepreneurial motivation will be weakened.

### Lean and Innovative Entrepreneurship

The concept of lean entrepreneurship was proposed by Silicon Valley scholar Ries in his book *Lean Entrepreneurship*, which is a description of a form of entrepreneurship. The process of entrepreneurship can be understood as putting a simple primary product into the market and analyzing its use feedback and demand in the market. In 1989, the researcher Krafcik of the Minimum Viable Product (MVP) used the “buffer” and “lean” to define different types of production systems. Among them, the lean production system is based on the lowest inventory to reduce costs and timely identify quality problems and correct them, thereby reducing error costs. Ultimately, the product quality is improved in the production process (Zainuddin and Halili, 2016).

**FIGURE 1:**
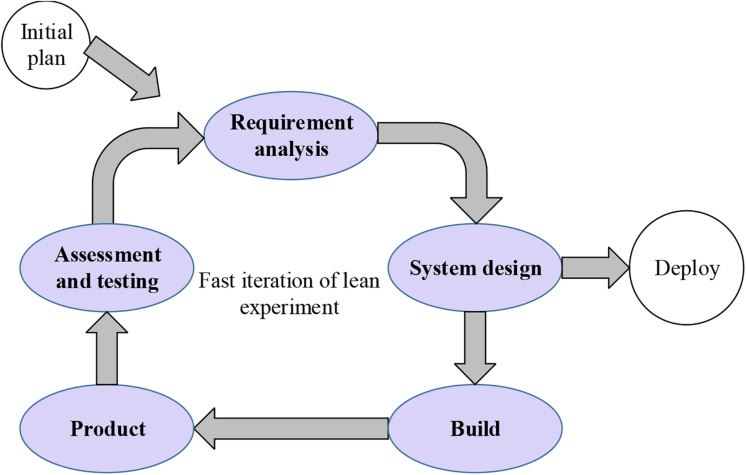
The cyclical process of lean entrepreneurship theory.

Womack et al. (1990), members of the Massachusetts Institute of Technology’s International Motor Vehicle Program, believed that lean production refers to streamlining manpower, equipment, and time as much as possible while creating value as many as possible. The core of lean thinking is to solve the actual problems of customers and produce products that meet the needs of customers. Besides that, while creating the practical application value of the product, the enterprise can obtain the benefits as much as possible (Lo and Hew, 2017). The innovative entrepreneurial theory based on lean thinking advocates the fastest cycle of product development, application measurement, and feedback recognition through the development of minimized products with lower costs, thereby obtaining the fastest recognition of products. Also, the product quality will be improved in the next product development and optimization. The specific process is shown in Figure 1.

In the process of innovation and entrepreneurship, only through repeated experiments and constant amendments can the progress of entrepreneurship be promoted. Lean entrepreneurship does not focus on a complete business plan. It is through trial and error of small products in the process of entrepreneurship; the plans are constantly changed according to requirements in the product update iteration, thereby flexibly and rapidly gaining innovative advantages. Lean entrepreneurship exists in production lines and various environments that require innovation. The more complex the entrepreneurial environment, the higher the demand for lean entrepreneurship.

Entrepreneurship education, as a framework containing many elements, has been analyzed by many scholars from the perspectives of education, teaching, and entrepreneurship. However, no consensus has yet been reached (Long et al., 2017; Song and Kapur, 2017; Srinivasan et al., 2018). In the feedback loop of lean entrepreneurship, each cycle represents a new lean iteration and progress. In the process of exploring the entrepreneurial education mode of colleges, this paper divided it into four modules to progressively deepen the entrepreneurial education for students. The first level is education link based on the elective course of entrepreneurship. The second level is traditional entrepreneurial education mainly based on teaching. The third level is entrepreneurship education through the teaching mode of the flipped classroom. The fourth level is continuous, iterative entrepreneurship education. At each level, each stage completes the cycle of lean entrepreneurship. In the process of conducting entrepreneurship education for students, regardless of whether they have a willingness to start a business, they must receive entrepreneurship education (Bhagat et al., 2016). As far as basic education for entrepreneurship is concerned, there has been a fundamental change in the provider and demand side. The “demand side” of entrepreneurship education has changed from students with entrepreneurial intentions to all students in colleges. The “provider” of entrepreneurship education will change the teaching purpose and method due to the change of “demand side.”

With the deepening development of entrepreneurship education in China’s colleges, under the combined effect of policy support and higher education system regulations, there has been a systematic mode for entrepreneurship education for college students. In the basic education stage of entrepreneurship, students first assume that they have the willingness to innovate and start a business. If the assumption is true, they follow the willingness to invest in the second stage of entrepreneurial skills education. If the assumption is not true, entrepreneurship education will no longer be continued. At the entrepreneurial skills education stage, students need to measure their entrepreneurial abilities and obtain the overall assessment of the knowledge from entrepreneurship education. At this stage, if they decide to continue to adhere to the idea of entrepreneurship after comprehensive consideration, they can enter the practical education stage of entrepreneurship education. If they give up the cultivation of entrepreneurial abilities, they can stop learning and develop other abilities (Foldnes, 2016). When students enter the stage of entrepreneurship practice education, they need to plan their start-up projects according to personal abilities and market environment. If the project evaluation is successful, it will enter the entrepreneurial incubation stage. After the student’s entrepreneurial project has passed the quasi-corporate management test, it means that it has successfully passed the start-up period.

### Flipped Classroom Entrepreneurship Education Mode Based on Deep Learning

The most direct understanding of the flipped classroom is to turn the traditional education mode of “teacher teaching, students completing homework” into the mode of promoting knowledge internalization of “allowing students complete autonomous learning of key knowledge theories outside the classroom and discussing the existing questions with teachers and students in class” (Sun et al., 2017; Zhai et al., 2017). This paper believes that the reform of the flipped classroom for the teaching mode provides students with more possibilities for autonomous learning and knowledge internalization. Students can find solutions to problems encountered during the pre-class learning process by themselves. They can gain knowledge in the process of solving problems, thereby deepening impressions. No matter what angle it is based on, the flipped classroom is designed to provide students with rich learning resources to complete the construction of the knowledge system through personal abilities and to acquire knowledge in a more targeted manner in the future study. It belongs to the result of deep learning. Deep learning is a process in which learners critically learn new ideas based on understanding learning and transfer existing knowledge to new situations to solve more complex problems. The teaching activities at each stage of the flipped classroom are shown in Table 1.

**TABLE 1:** Teaching activities at each stage of the flipped classroom.

Teaching links	Teacher	Student	Evaluate	Assessment index
Before class	a. To determine learning resources and design learning tasks according to teaching objectives and contents b. Release learning tasks and learning resources based on the platform c. Collect, organize, and analyze pre-class questions	a. Use learning resources to learn and complete related learning tasks b. Problems encountered in the process of submitting learning on the platform c. Participate in the discussion of issues	The teacher evaluates the whole team and the team leader evaluates the members	a. Whether to complete the learning task b. Whether to submit questions c. Test scores d. Whether to participate in the discussion
In class	a. Arrange a knowledge point test b. Arrange speeches and exhibitions c. Arrange a discussion d. Answer the difficulties and key points e. Sum up	a. Classroom tests b. Speech and exhibition c. Discussion and cooperation d. Take notes e. Other issues	Teachers evaluate the whole group, members evaluate each other, and groups evaluate each other	a. Test scores b. Presentation and performance c. Achievements in discussion and cooperation d. Other classroom performance
After class	a. Arrange difficulties and key tests b. Assign extended learning tasks	a. Complete section testing b. Complete the task of expanding learning	Teachers evaluate all, group leaders evaluate members, students evaluate teachers, and teaching reflection	a. Test scores b. Expand task performance

The autonomous learning stage before class is mainly to build a knowledge framework. However, it is difficult to achieve optimal deep learning only by students’ autonomous learning. For most students, the purpose of pre-class learning is to promote deep cognition and provide learning materials for capable students to carry out further deep learning. In the pre-class learning stage, teachers should leave some space for students to carry out deep learning. They should focus on the basic content and guide students to dig and think on their own (Ryan and Reid, 2016; Aidinopoulou and Sampson, 2017; Wu W. et al., 2019). In the classroom learning stage, teachers act as organizers to guide and promote students to think deeply about knowledge. At the same time, corresponding solutions are developed for the content that is difficult to grasp before class to achieve a comprehensive grasp of knowledge. Deep learning in the flipped classroom should be mainly realized through classroom activities. The personal participation of students can promote the achievement of deep learning goals to a certain extent. The teachers can arrange learning goals in all links of the flipped classroom to motivate students’ deeper learning motivation and increase learning investment. For lean entrepreneurship education in colleges, the basic education of entrepreneurship based on the flipped classroom emphasizes independent thinking from the purpose of education, attaches importance to students’ learning experience, and practices what they have learned. As a place of thought interaction, the classroom integrates traditional entrepreneurial extracurricular activities into the entrepreneurial basic classroom. By quickly iterating the teaching content, it helps students develop deep thinking and teamwork skills, thereby cultivating students’ innovative entrepreneurial spirit.

Examination results are a conventional method for evaluating teaching effects. The results are affected by many factors, but the result analysis based on the Markov chain can effectively avoid the interference caused by differences (Lundin et al., 2018; Wu Y. et al., 2019; Zainuddin and Perera, 2019). In this study, three-stage tests are conducted in teaching activities. To objectively evaluate the impact of the flipped classroom teaching mode on the results of deep learning, the Markov analysis is performed for the data in each stage test. The results are divided into five levels: ≥90 is divided into level A, 80–89 is divided into level B, 70–79 is divided into level C, 60–69 is divided into level D, and ≤60 is divided into level E. According to the five levels of the score, it is assumed that there are *n* students in total. Then, there will be *n*_*i*_ students in the *i*th level and *i* = (1,2,3,4,5). The initial vector of the data distribution can be expressed as follows:

(1) $$\Pi_{0}=\left(\frac{n_{0}}{n},\frac{n_{1}}{n},\ldots ,\frac{n_{S-1}}{n}\right)$$

The probability transition matrix *p* of the first test result to the second test result can be expressed as follows:

(2) $$p=\left(\begin{array}{c} \frac{n_{0}}{n_{0}},\frac{n_{1}}{n_{0}},\ldots ,\frac{n_{04}}{n_{0}}\\ \frac{n_{10}}{n_{1}},\frac{n_{11}}{n_{1}},\ldots ,\frac{n_{14}}{n_{1}}\\ \ldots \\ \frac{n_{40}}{n_{4}},\frac{n_{41}}{n_{4}},\ldots ,\frac{n_{44}}{n_{4}} \end{array}\right)$$

(3) $$\sum_{j=0}^{4}p_{i\,j}=1,0\geq p_{i\,j}\geq 1,\left(i,j=0,1,2,3,4\right)$$

(4) $$\Pi_{n}=\Pi_{0}\,p$$

In the Markov analysis, it can be obtained from the ergodic theorem, *X* = (*x*_1_, *x*_2_, *x*_3_, *x*_4_, *x*_5_ ≠ 0 is the steady distribution of the state *R*(*t*). According to the *X* result (0.221, 0.583, 0.187, 0.009, 0.000), five levels are assigned, and the teaching effect *E* after three times of flipped classroom teaching can be expressed as shown below:

(5) E=0.221×95+0.583×85+0.187×75+0.009×65+0.000×0=85.16

Changes in the test results of students at various stages can indicate that learning is progressing or regressing. The change of learning status can be expressed by the level change, which reflects the teaching effect. The Markov chain progression matrix equation can be expressed as follows:

(6) $$L={\left(L_{i\,j}\right)}_{5\times 5}={\left|\frac{{\left(i-j\right)}^{3}\,n_{i\,j}}{n_{i}}\right|}_{5\times 5}$$

### Research Sample and Methods

In this study, 348 students who participated in entrepreneurial basic education among the sophomores in the first semester of the academic year 2018–2019 of a college in Shanghai were selected as samples. According to the different stages of students’ participation in entrepreneurial basic education, they were divided into a research group (flipped classroom) and a control group (traditional education). The teaching effect of the two groups of students was compared and analyzed using pre-sample and post-sample tests. Through empirical research, the influence of basic education for entrepreneurship based on the flipped classroom on college students’ entrepreneurial intentions was verified (Wu and Song, 2019). Furthermore, the application effect of the basic education mode of lean entrepreneurship in colleges was explored. The research used data from two entrepreneurial basic education courses taught by the same teacher at the same time to avoid the impact of other factors on teaching effect. In addition, the effect of the flipped classroom teaching mode on improving students’ deep learning and autonomous learning ability needs to be tested.

#### Research Variables and Measurement Methods

**FIGURE 2:**
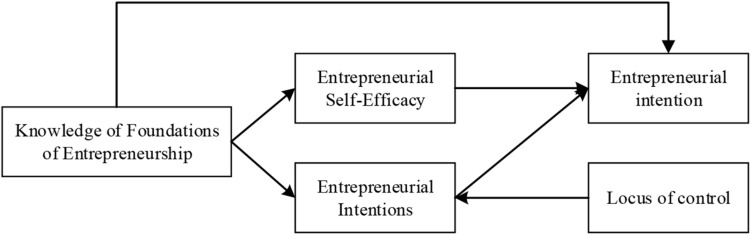
Influencing factor model of college students’ entrepreneurial intentions.

It is necessary to construct a model of influencing factors for college students’ entrepreneurial intentions, including two types of subjective variables, namely, intention variables and attitude variables. Specifically, there is the knowledge of foundations of entrepreneurship, entrepreneurial self-efficacy, and entrepreneurial intentions. By constructing the influence model of college students’ entrepreneurial intention factors, the intermediary and the adjustment effects of the model are analyzed and verified. The model is shown in Figure 2. The variable selection and measurement in this study are from a scale that has been widely used in this field to ensure the validity of the measurement results (Chen, 2019).

#### Questionnaire Distribution and Recovery

Before and after carrying out the research, the questionnaire was distributed to students through the Wenjuanxing platform, and the data were collected through online surveys. The teacher provided the QR code of the online questionnaire to the students in the classroom, and the students scanned the code to answer. The research group distributed a total of 218 questionnaires and recovered 212 valid questionnaires, with a recovery rate of 97.2%. The control group distributed 130 questionnaires and recovered 121 valid questionnaires, with a recovery rate of 93.1%. All valid questionnaire data were exported to CSV format, and SPSS 21.0 software was used for statistical analysis.

## Results

### Effects of Lean Entrepreneurship Education for College Students

**FIGURE 3:**
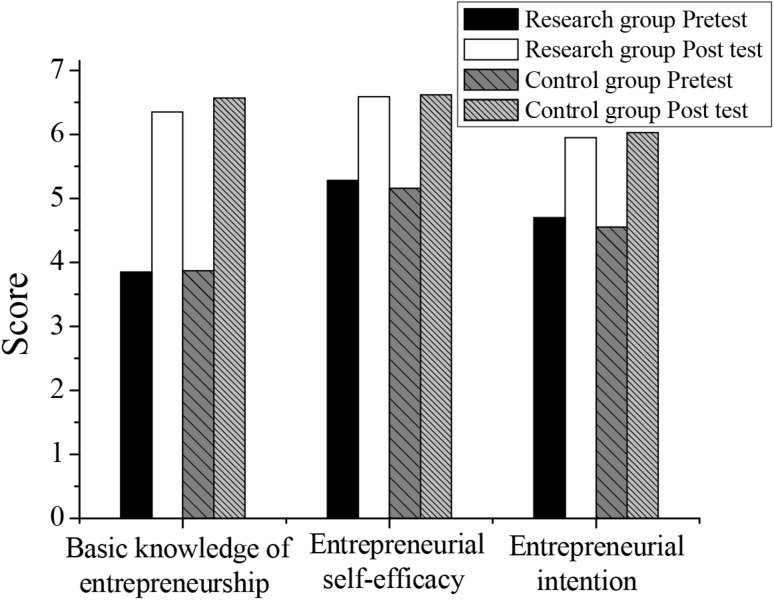
Comparison of the descriptive statistical results of two groups of students.

The results of the survey on the basic knowledge of entrepreneurship, entrepreneurial self-efficacy, and entrepreneurial intention of students in the research group and the control group are shown in Table 2 and Figure 3. It can be seen that after receiving the entrepreneurship education, the scores of the three aspects of students in the basic knowledge of entrepreneurship, entrepreneurial self-efficacy, and entrepreneurial intention have significantly improved compared with those in the same group before education. Thus, entrepreneurship education carried out by colleges has a positive effect on improving students’ entrepreneurial awareness and establishing a good entrepreneurial concept. Through theoretical explanations and case analysis, students’ entrepreneurial ability can be cultivated. Before the course, there is no difference between the pretest scores of the two groups (*P* > 0.05). After the course, there is no difference between the posttest scores of the two groups (*P* > 0.05). It can be confirmed that, based on the flipped classroom education mode of halving the actual number of lectures by teachers, the effect of basic education on entrepreneurship for students is not different from the traditional teaching effect.

**TABLE 2:** Descriptive statistics of students in the research and the control groups.

Research variables	Research group		Research group		Control group		Control group	
	pretest (*n* = 212)		posttest (*n* = 212)		pretest (*n* = 121)		posttest (*n* = 121)	
	Mean	SD	Mean	SD	Mean	SD	Mean	SD
Basic knowledge of entrepreneurship	3.85	1.56	6.35	1.60	3.87	1.90	6.57	1.88
Entrepreneurial self-efficacy	5.28	1.86	6.59	1.73	5.16	2.11	6.62	1.97
Entrepreneurial intention	4.70	1.82	5.95	1.87	4.55	1.96	6.03	2.01

Before and after the students in the research group participate in the entrepreneurship course, the score of the basic knowledge of entrepreneurship is 3.85 and 6.35 (*t* = 18.897, *P* < 0.001), respectively. The score of the entrepreneurial self-efficacy is 5.28 and 6.59 (*t* = 11.243, *P* < 0.001), respectively. The score of entrepreneurial intention is 4.70 and 5.95 (*t* = 12.036, *P* < 0.001), respectively. It can be confirmed that the education mode based on the flipped classroom has a significant effect on the basic knowledge of entrepreneurship of college students and has a significant impact on entrepreneurial self-efficacy and entrepreneurial intention. Before and after the students in the control group participated in the entrepreneurship course, the score of the basic knowledge of entrepreneurship is 3.87 and 6.57 (*t* = 13.290, *P* < 0.001), respectively. The score of the entrepreneurial self-efficacy is 5.16 and 6.62 (*t* = 6.551, *P* < 0.001), respectively. The score of entrepreneurial intention is 4.55 and 6.03 (*t* = 6.732, *P* < 0.001), respectively.

### The Role of Flipped Classroom in Entrepreneurship Education for Promoting Deep Learning

**FIGURE 4:**
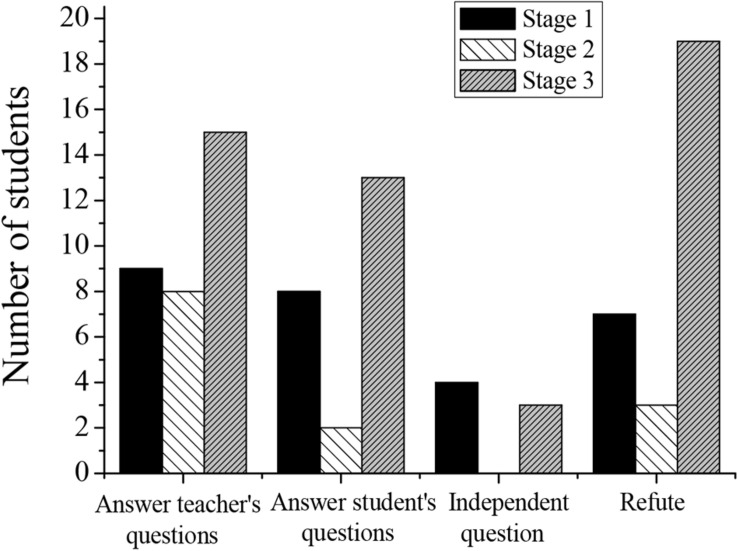
The deep cognition of a three-stage classroom record of students.

**FIGURE 5:**
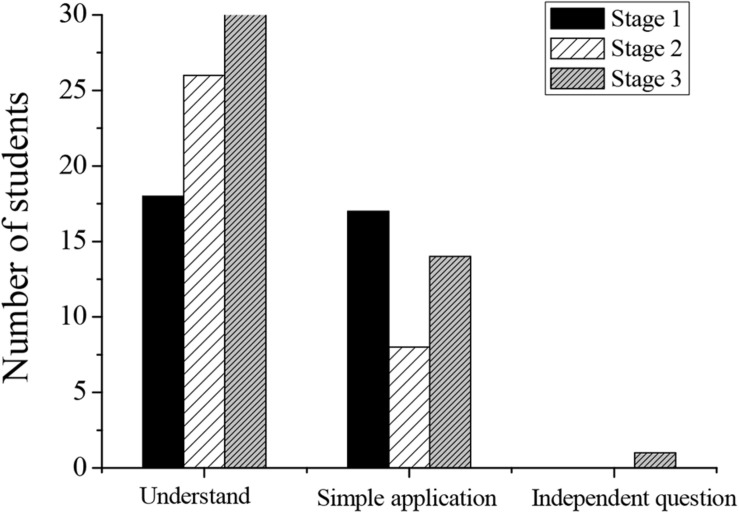
Deep cognition of the three-stage text tasks of students.

In the process of entrepreneurship education, achieving effective teaching results is the ultimate goal of entrepreneurship education. Furthermore, this paper applies the teaching mode of flipped classroom to promote students’ deep learning in the teaching process and implement entrepreneurship education. The state results of student deep learning in this study mainly come from task text analysis. The pretest tasks (reflecting students’ initial level of deep learning in learning content), classroom learning tasks (reflecting students’ deep learning level in the classroom), and posttest tasks (reflecting students’ deep learning state after flipped classroom teaching mode) are designed. This paper focuses on the deep learning state of 35 students in a class in the research group. At present, it is believed that deep learning should achieve deep thinking in both the target and the cognition dimensions. If both dimensions are in a shallow state and fail to achieve deep learning, it is considered to be in the least ideal learning state at this time. If one dimension is in the deep state and the other dimension is in the shallow state, it is considered to be in a transition state from shallow to deep learning. If both dimensions reach a deep state, it is the ideal deep learning state. This study counts the results of text analysis and classroom record on the target and the cognition dimensions. The statistical results are shown in Tables 3, 4 and Figures 4, 5.

**TABLE 3:** Three-stage classroom record of students in a flipped classroom mode.

Deep learning state		Stage 1		Stage 2		Stage 3	
		Proportion	Deep	Proportion	Deep	Proportion	Deep
		reached	cognition	reached	cognition	reached	cognition
Low-level motivation	Answer teacher’s questions	23/35	9/23	27/35	8/27	29/35	15/29
	Answer student’s questions	15/35	8/15	4/35	2/4	18/35	13/18
High-level motivation	Independent question	7/35	4/7	1/35	0/1	7/35	3/7
	Refute	14/35	8/14	6/35	3/6	20/35	10/20

**TABLE 4:** Analysis of the three-stage text tasks of the students in a flipped classroom mode.

Target		Stage 1		Stage 2		Stage 3	
		Target	Deep	Target	Deep	Target	Deep
		achievement	cognition	achievement	cognition	achievement	cognition
Low-order target	Memory	–	–	–	–	–	–
	Understand	34/35	18/34	28/35	26/28	34/35	30/34
	Simple application	18/25	17/18	9/35	8/9	18/25	14/18
High-order target	Independent question	0	0	0	0	1/35	1/35

In the autonomous learning stage before class, the main task was to master the basic knowledge. Students needed to consult information and learn relevant knowledge according to their ideas. However, the learning effect at this stage was affected by personal habits and ability levels. According to statistical results, it was found that the number of students who can achieve both the deep cognition and the high-order target in the three stages of pre-class learning is small. Based on the flipped classroom mode, the number of people who reached the “understanding” level of the text target task is the largest, and 52.9% of them can reach the deep cognition at the same time. The low-order target of the first stage is the same as that of the third stage, and 56% of students can reach the target. The number of “simple application” at the second stage decreases significantly, only 25.7%. In the first two stages, no student is able to achieve the “comprehensive application” level of the high-order target. Only one person can achieve in the third stage, which means that the achievement of the high-order target still needs to be strengthened.

## Discussion

Entrepreneurship is a universal activity in the market economy, and research on entrepreneurship has developed rapidly since the 1990s. In the context of China’s explicit promotion of entrepreneurship for college students, colleges have gradually begun to provide corresponding support and skills training for college entrepreneurs. They should not only teach students the necessary skills to start a business but also pass on how to strengthen their entrepreneurial awareness. College entrepreneurship can avoid wasting educational resources. The production and development are driven through high-tech industries, thereby promoting China’s transformation to a knowledge-intensive economic mode. Entrepreneurial motivation is the product of the interaction of individuals and external factors. As a personal trait, the locus of control has an important influence on entrepreneurial behavior and the improvement of basic quality. Entrepreneurship education can improve the basic quality of entrepreneurs and cultivate specialized talents from all aspects of disciplines and specialties. As the innovation and entrepreneurship education develops, the basic entrepreneurial education, as the core of college students’ entrepreneurship education and teaching activities, is characterized by standardization and implementation.

The concept of “lean entrepreneurship” reflects the application of lean thinking in the field of innovation and entrepreneurship. The introduction of “lean entrepreneurship” into the field of entrepreneurship education is mainly a further exploration of the “development–measurement–learning” method (Chen, 2019; Shen and Ho, 2020). The basic education of entrepreneurship based on lean entrepreneurial thinking is no longer limited to students who are willing to start a business, and the entrepreneurial quality education is implemented for all students. As far as basic education for entrepreneurship is concerned, there has been a fundamental change in supply and demand. The “demand side” of entrepreneurship education has changed from students with entrepreneurial intentions to all students in colleges. The “provider” of entrepreneurship education will change the teaching purpose and the method due to the change of the “demand side.” This paper first introduces lean entrepreneurial thinking into the field of entrepreneurship education. In the process of implementing entrepreneurial basic education, the education mode of the flipped classroom is integrated to realize the reversal of the teaching process. It helps to divert students’ thinking, promote autonomous learning, cultivate high-order thinking, and creatively deal with problems. The flipped classroom mode includes three links before, during, and after the class. The flipped classroom based on deep learning gives students more autonomy and can enhance the achievement of the high-order target.

In this study, students who participated in entrepreneurial basic education among the sophomores in the first semester of the academic year 2018–2019 of a college in Shanghai were selected as samples. Through empirical research, the influence of basic education for entrepreneurship based on the flipped classroom on college students’ entrepreneurial intentions was verified. Furthermore, the application effect of the basic education mode of lean entrepreneurship in colleges was explored. The entrepreneurship education carried out by colleges has a positive effect on improving students’ entrepreneurial awareness and establishing a good entrepreneurial concept. Through theoretical explanations and case analysis, students’ entrepreneurial ability can be cultivated. Before the course, there was no difference between the pretest scores of the two groups (*P* > 0.05). After the course, there was no difference between the posttest scores of the two groups (*P* > 0.05). It can be confirmed that, based on the flipped classroom education mode of halving the actual number of lectures by teachers, the effect of basic education on entrepreneurship for students is not different from the traditional teaching effect. The innovation of this research lies in the first introduction of lean entrepreneurship into the field of entrepreneurship education and the construction of a “lean entrepreneurship education mode” from the perspective of individual student development (Wu et al., 2020). Also, taking the sample colleges as an example, the practical application effects and advantages of the lean entrepreneurship education mode are explained. This model aims at creating student value, takes action priority as well as scientific trial and error as the principle of conduct, takes the tolerable loss as the basis for decision-making, and takes rapid lean iteration as the guiding ideology. It can effectively guide the overall deployment of entrepreneurship education and the development of entrepreneurship basic education in colleges, providing an effective action paradigm and method for Chinese colleges to deepen the reform of entrepreneurship education.

## Conclusion

Lean entrepreneurship education mode takes lean iteration as well as scientific trial and error as the principle. It aims to achieve the purpose of creating student value and it is conducive to promoting the development of entrepreneurship education in China. Based on lean entrepreneurship, the teaching mode of the flipped classroom is integrated. Through the flipped teaching process, the goal of deep learning can be achieved ideally. In the process of basic entrepreneurship education for college students, the two principles of high participation and high-order target have been taken into consideration. This study provides a new theoretical framework for the development of entrepreneurship education, and it has important practical significance for improving the effectiveness of entrepreneurship education. Due to the limitation of ability level, the evaluation of deep learning in this research is still in the preliminary exploration stage. Therefore, the consideration of some dimensions and indicators is not comprehensive enough, and it should be further improved in future research.

## Data Availability Statement

The raw data supporting the conclusions of this article will be made available by the authors, without undue reservation.

## Ethics Statement

The studies involving human participants were reviewed and approved by the East China Normal University Ethics Committee. The patients/participants provided their written informed consent to participate in this study.

## Author Contributions

YQ and YS made together for the manuscript. All authors contributed to the article and approved the submitted version.

## Conflict of Interest

The authors declare that the research was conducted in the absence of any commercial or financial relationships that could be construed as a potential conflict of interest.

## References

[B1] Aidinopoulou V. Sampson D. G. (2017). An action research study from implementing the flipped classroom model in primary school history teaching and learning. *J. Educ. Technol. Soc.* 20 237–247.

[B2] Bhagat K. K. Chang C. N. Chang C. Y. (2016). The impact of the flipped classroom on mathematics concept learning in high school. *J. Educ. Technol. Soc.* 19 134–142.

[B3] Blank S. (2016). Why the lean start-up changes everything. *Harv. Bus. Rev.* 91 0–72.

[B4] Chen M. (2019). The impact of expatriates’ cross-cultural adjustment on work stress and job involvement in the high-tech industry. *Front. Psychol.* 10:2228. 10.3389/fpsyg.2019.02228 31649581 PMC6794360

[B5] Euchner J. (2018). The genesis and future of lean startup: an interview with steve blank: steve blank talks with jim euchner about the emergence of the lean startup movement and its application in corporations and government. *Res. Technol. Manag.* 61:15 10.1080/08956308.2018.1495963

[B6] Foldnes N. (2016). The flipped classroom and cooperative learning: evidence from a randomised experiment. *Active Learn. Higher Educ.* 17 39–49. 10.1177/1469787415616726

[B7] Frederiksen D. L. Brem A. (2017). How do entrepreneurs think they create value? A scientific reflection of Eric Ries’ Lean Startup approach. *Int. Entrep. Manag. J.* 13 169–189. 10.1007/s11365-016-0411-x

[B8] Harms R. Schwery M. (2020). Lean startup: operationalizing lean startup capability and testing its performance implications. *J. Small Bus. Manag.* 58 200–223. 10.1080/00472778.2019.1659677

[B9] Hwang S. Shin J. (2019). Using lean startup to power organizational transformation: creating an internal division that implemented concepts from lean startup helped a consumer electronics firm foster an entrepreneurial mindset among employees. *Res. Technol. Manag.* 62 40–49. 10.1080/08956308.2019.1638224

[B10] Lichtenthaler U. (2020). Agile innovation: the complementarity of design thinking and lean startup. *Int. J. Serv. Sci. Manag. Eng. Technol.* 11 157–167. 10.4018/ijssmet.2020010110

[B11] Llamas F. F. J. Fernández R. J. C. (2018). La metodología lean startup: desarrollo y aplicación para el emprendimiento. *Revista EAN* 84 79–95.

[B12] Lo C. K. Hew K. F. (2017). A critical review of flipped classroom challenges in K-12 education: possible solutions and recommendations for future research. *Res. Pract. Technol. Enhan. Learn.* 12:4. 10.1186/s41039-016-0044-2 PMC6302872 30613253

[B13] Long T. Cummins J. Waugh M. (2017). Use of the flipped classroom instructional model in higher education: instructors’ perspectives. *J. Comput. Higher Educ.* 29 179–200. 10.1007/s12528-016-9119-8

[B14] Lundin M. Rensfeldt A. B. Hillman T. (2018). Higher education dominance and siloed knowledge: a systematic review of flipped classroom research. *Int. J. Educ. Technol. Higher Educ.* 15:20.

[B15] Lüthje C. Franke N. (2003). The ‘making’of an entrepreneur: testing a model of entrepreneurial intent among engineering students at MIT. *R&d Manag.* 33 135–147. 10.1111/1467-9310.00288

[B16] Mansoori Y. (2017). Enacting the lean startup methodology: the role of vicarious and experiential learning processes. *Int. J. Entrepren. Behav. Res.* 23 812–838. 10.1108/ijebr-06-2016-0195

[B17] Miao C. Qian S. Humphrey R. H. (2019). The challenges of Lean management research and practice in the field of entrepreneurship: the roles of IO psychology theories and IO psychologists. *Industr. Organ. Psychol.* 12 260–263. 10.1017/iop.2019.46

[B18] Ryan M. D. Reid S. A. (2016). Impact of the flipped classroom on student performance and retention: a parallel controlled study in general chemistry. *J. Chem. Educ.* 93 13–23. 10.1021/acs.jchemed.5b00717

[B19] Shen C.-W. Ho J.-T. (2020). Technology-enhanced learning in higher education: a bibliometric analysis with latent semantic approach. *Comput. Hum. Behav.* 104 106177 10.1016/j.chb.2019.106177

[B20] Song Y. Kapur M. (2017). How to flip the classroom–“productive failure or traditional flipped classroom” pedagogical design? *J. Educ. Technol. Soc.* 20 292–305.

[B21] Souza J. (2020). Blended learning: study of a formative assessment in the flipped classroom model. *Arch. Bus. Res.* 8 1–7. 10.14738/abr.82.7772

[B22] Srinivasan S. Gibbons R. E. Murphy K. L. Raker J. (2018). Flipped classroom use in chemistry education: results from a survey of postsecondary faculty members. *Chem. Educ. Res. Pract.* 19 1307–1318. 10.1039/c8rp00094h

[B23] Sun J. C. Y. Wu Y. T. Lee W. I. (2017). The effect of the flipped classroom approach to OpenCourseWare instruction on students’ self-regulation. *Br. J. Educ. Technol.* 48 713–729. 10.1111/bjet.12444

[B24] Womack J. Jones D. Roos D. (1990). *The Machine that Changed the World*, New York, NY: Rawson Associates.

[B25] Wu W. Wang H. Zheng C. Wu Y. J. (2019). Effect of narcissism, psychopathy, and machiavellianism on entrepreneurial intention—the mediating of entrepreneurial self-efficacy. *Front. Psychol.* 10:360. 10.3389/fpsyg.2019.00360 30846958 PMC6393355

[B26] Wu Y. Song D. (2019). Gratifications for social media use in entrepreneurship courses: learners’ perspective. *Front. Psychol.* 10:1270. 10.3389/fpsyg.2019.01270 PMC6555126 31214081

[B27] Wu Y. Wu T. Li Y. (2019). Impact of using classroom response systems on students’ entrepreneurship learning experience. *Comput. Hum. Behav.* 92 634–645. 10.1016/j.chb.2017.08.013

[B28] Wu Y. J. Liu W.-J. Yuan C.-H. (2020). A mobile-based barrier-free service transportation platform for people with disabilities. *Comput. Hum. Behav.* 107:105776 10.1016/j.chb.2018.11.005

[B29] Yang X. Sun S. L. Zhao X. (2019). Search and execution: examining the entrepreneurial cognitions behind the lean startup model. *Small Bus. Econom.* 52 667–679. 10.1007/s11187-017-9978-z

[B30] Yordanova Z. B. (2017). Knowledge transfer from lean startup method to project management for boosting innovation projects’ performance. *Int. J. Technol. Learn. Innov. Dev.* 9 293–309.

[B31] Zainuddin Z. Halili S. H. (2016). Flipped classroom research and trends from different fields of study. *Int. Rev. Res. Open Distrib. Learn.* 17 313–340.

[B32] Zainuddin Z. Perera C. J. (2019). Exploring students’ competence, autonomy and relatedness in the flipped classroom pedagogical model. *J. Furth. Higher Educ.* 43 115–126.

[B33] Zhai X. Gu J. Liu H. Jyh-Chong L. Chin-Chung T. (2017). An experiential learning perspective on students’ satisfaction model in a flipped classroom context. *J. Educ. Technol. Soc.* 20 198–210.

